# Temporal Uncoupling between Energy Acquisition and Allocation to Reproduction in a Herbivorous-Detritivorous Fish

**DOI:** 10.1371/journal.pone.0150082

**Published:** 2016-03-03

**Authors:** Francisco Villamarín, William E. Magnusson, Timothy D. Jardine, Dominic Valdez, Ryan Woods, Stuart E. Bunn

**Affiliations:** 1 Australian Rivers Institute - ARI, Griffith University, Brisbane, Australia; 2 Coordenação de Pesquisas em Biodiversidade, Instituto Nacional de Pesquisas da Amazônia - INPA, Manaus, Brazil; 3 Programa Ciência Sem Fronteiras, Conselho Nacional de Desenvolvimento Científico e Tecnológico - CNPq, Brasilia, Brazil; 4 School of Environment and Sustainability, University of Saskatchewan, Saskatoon, Canada; Universidad de la Republica, URUGUAY

## Abstract

Although considerable knowledge has been gathered regarding the role of fish in cycling and translocation of nutrients across ecosystem boundaries, little information is available on how the energy obtained from different ecosystems is temporally allocated in fish bodies. Although in theory, limitations on energy budgets promote the existence of a trade-off between energy allocated to reproduction and somatic growth, this trade-off has rarely been found under natural conditions. Combining information on RNA:DNA ratios and carbon and nitrogen stable-isotope analyses we were able to achieve novel insights into the reproductive allocation of diamond mullet (*Liza alata*), a catadromous, widely distributed herbivorous-detritivorous fish. Although diamond mullet were in better condition during the wet season, most reproductive allocation occurred during the dry season when resources are limited and fish have poorer body condition. We found a strong trade-off between reproductive and somatic investment. Values of *δ*^13^C from reproductive and somatic tissues were correlated, probably because *δ*^13^C in food resources between dry and wet seasons do not differ markedly. On the other hand, data for *δ*^15^N showed that gonads are more correlated to muscle, a slow turnover tissue, suggesting long term synthesis of reproductive tissues. In combination, these lines of evidence suggest that *L*. *alata* is a capital breeder which shows temporal uncoupling of resource ingestion, energy storage and later allocation to reproduction.

## Introduction

Linkages among habitats and the flux of matter across ecosystem boundaries have important implications for biomass production of animals and plants [1–4]. As part of spawning movements and migrations, many species of fish cross ecosystem boundaries. For example, in the wet-dry tropics, fish that typically live in river channels, waterholes and estuaries during the dry season move onto floodplains during the wet season, where they take advantage of abundant resources. Subsequently, as flood waters recede, many fish disperse back to waterholes, river channels and estuaries. It has been well documented that fish movements may transfer nutrients and aquatic production [3,5], but information on how this production contributes to individual energy allocation and reproductive investment is scarce (but see [4]). Much of this energy might be obtained from floodplain productivity.

Floodplains provide large amounts of high quality resources to consumers [4,6], with higher production of macrophytes, phytoplankton and attached algae during periods of inundation [7]. In general, abundant food resources support earlier maturation and higher fecundity in fish (see [8]). However, it is not well understood how the temporally abundant subsidies from floodplains are allocated to consumer tissues. According to life-history theory, allocation to reproduction results in a trade-off with somatic growth or survival because of limitations of the resource budget [9–14]. However, trade-offs between somatic growth and reproduction have rarely been observed under natural conditions [14,15].

Organisms differ in their allocation of resources to reproduction. Diadromous species engage in energetically expensive migrations for spawning and use surplus energy stored from previous periods to fuel reproductive output [8,16–18], a strategy known as ‘capital breeding’ [19–21]. Conversely, the coupling of the reproductive cycle with temporally abundant resources during the breeding period is characterized as ´income breeding´. On floodplains with short inundation periods (~2 months) in the Australian wet-dry tropics, the development of reproductive tissues was fueled by resources available at the time of spawning in the herbivorous fish, *Nematalosa come* [4]. Therefore, the reproductive cycle of this ‘income-breeding’ fish, not known to undertake upstream spawning migrations [22], is coupled with the temporally abundant resources from floodplains, despite the short inundation duration.

Another common species in the Australian wet-dry tropics, the diamond mullet (*Liza alata*), is a catadromous fish broadly distributed in the Indo-west-central Pacific. This herbivorous-detritivorous fish breeds in estuaries and spawns large numbers of pelagic non-adhesive eggs [23], as do most of the other members of the Mugilidae [24]. Juveniles recruit in estuarine areas from where they move up river. Although information on reproduction of this species is scarce, Bishop [23] found higher values of gonadosomatic index (GSI) during the early-wet season in the Alligator Rivers region and suggested that spawning migrations must occur during the wet season, which is the only time when seasonally isolated water bodies are connected to the sea for more than 4 months. It is unknown whether this species is a capital or an income breeder. If resources from floodplains are instantly allocated to reproduction, then greater growth of reproductive tissues is expected during the wet season. On the other hand, if mullet carry out spawning migrations to the sea during the wet season, they must have enough stored resources to synthesize gonads before flooding begins and we would expect higher reproductive investment during the dry season. In this case, reproductive investment would be temporally uncoupled from resource availability from floodplains.

Most studies under natural conditions have relied on morphological estimates of reproductive investment, such as total clutch mass, the number of young in a clutch and frequency of clutches (see [14]). However, besides the limitations on quantifying fecundity or reproductive effort based on counting fish eggs [8], those estimates do not express instantaneous growth of reproductive or somatic tissues. An alternative method, RNA:DNA ratio of a cell, is a biochemical indicator of recent growth in aquatic organisms [25–28]. This is because the amount of DNA present in a cell remains relatively constant, whereas RNA concentrations vary in proportion to protein synthesis [29–31]. Thus, by quantifying the ratio of these nucleic acids in the cells of fish it is possible to estimate the instantaneous investment in growth of different tissues. This can allow inferences as to whether the fish was investing in somatic or reproductive growth at the time of capture. Another method, stable-isotope analysis (SIA) has become a common tool for investigating trophic interactions and energy pathways in food webs [32–35] and can be used to determine the origin of resources in reproductive tissues [4]. Isotope values of vertebrate tissues depend in part on the isotopic turnover rates, with liver having a shorter half-life than muscle [36–39]. Half-lives of carbon and nitrogen in metabolically active fish tissues, such as liver, range in the order of days to weeks, while structural tissues, such as muscle, show half-lives in the range of weeks to months [39–41]. Therefore, theoretically, liver has the potential to provide more recent dietary information. Comparing isotope ratios of these fast and slow turnover tissues with those of gonads may be useful to indicate whether gonads were formed using resources available at the time of capture or a few months in advance [4].

Here we combine information on RNA:DNA ratios and stable-isotope analyses to achieve novel insights into the reproductive allocation strategies of diamond mullet. We analysed RNA:DNA and ratios of stable isotopes of carbon (*δ*^13^C) and nitrogen (*δ*^15^N) in multiple tissues of this fish to estimate the temporal importance of different aquatic habitats as a subsidy for the synthesis of somatic and reproductive tissues. Specifically, we asked the following questions: 1) When do mullet allocate energy resources for reproductive and somatic growth? 2) Is there a trade-off between reproductive and somatic investment? 3) How different are short- and long-term turnover tissues in terms of carbon and nitrogen stable isotopes?

## Materials and Methods

### Study area

**Fig 1:**
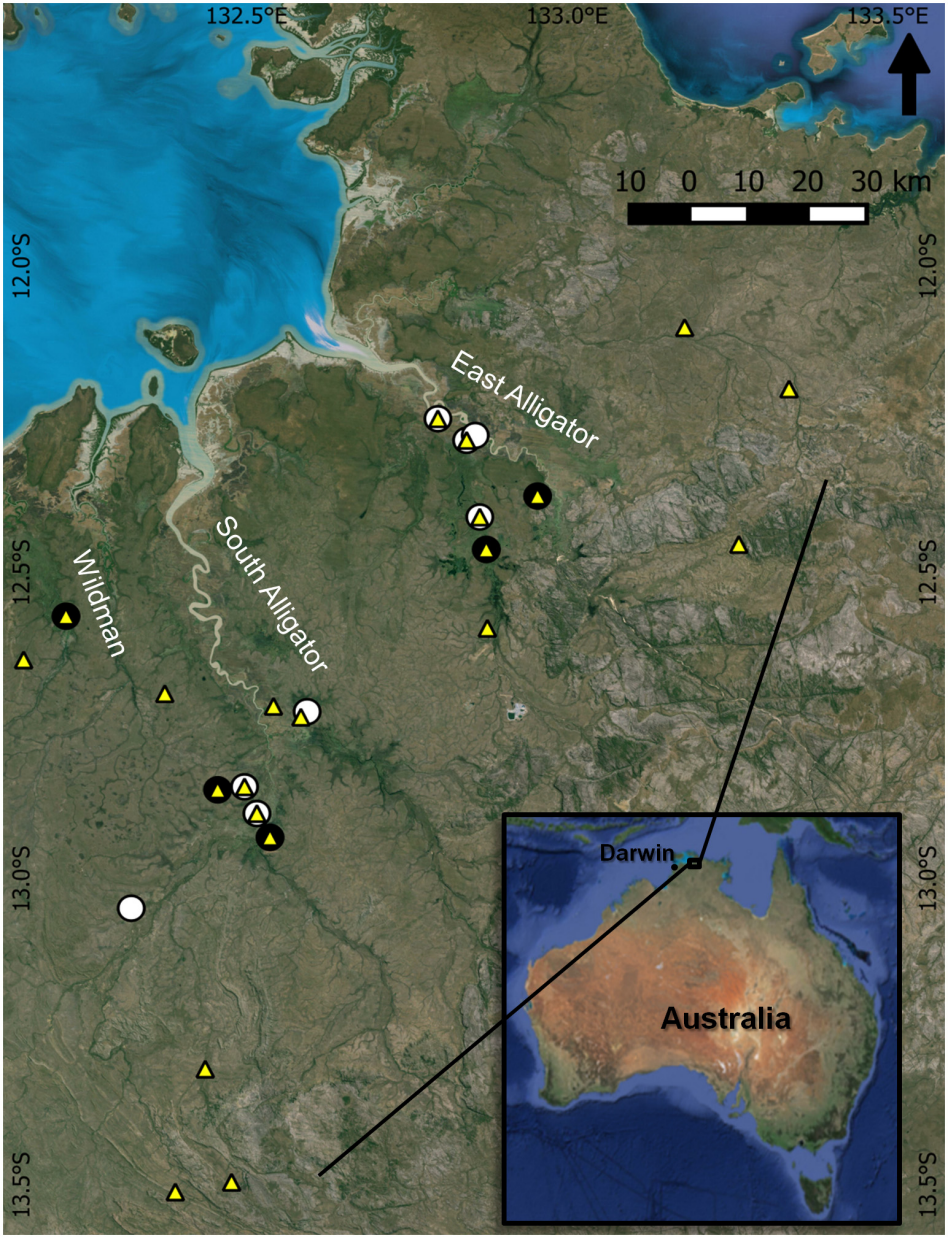
Study area. Location of mullet sampling sites during dry and wet seasons (black and white dots, respectively) in the Alligator Rivers region—Northern Territory, Australia. Yellow triangles represent locations where primary sources were collected.

This study was undertaken within the limits of Kakadu National Park, a protected area located in the Alligator Rivers region in the Northern Territory, Australia (Fig 1). This region is situated about 150 km east of the city of Darwin and is part of a bio-geographical region known as the Australian wet-dry tropics. The most conspicuous climatic characteristic of this region is the presence of a warm dry season and a warm-humid wet season [42,43]. The dynamic hydrology drives most ecosystem processes and structure, including primary productivity and subsidies to food webs through fish movements [4,44].

One fourth of the area of the Australian wet-dry tropics is comprised of floodplain wetlands [44]. These ecosystems experience extensive seasonal inundations and high river flows during the wet season between November and April. As rainfall decreases during the dry season, flows are reduced to zero in most rivers and waterbodies are contracted and isolated [45,46]. During this period, creeks and floodplain areas dry out except for a few permanent swamps and lagoons, known locally as billabongs or waterholes [47]. The Alligator Rivers region area is covered by water during March and April, which recedes to approximately 25%–30% of its maximum extent by August and September. It takes about 5 months to reduce to 50% of the maximum recorded flooded area in a given wet season [48]. Tidal influence extends for 70–90 km along the major rivers [47].

Permission to access biological resources in a commonwealth area for non-commercial purposes was provided by the Australian Government. Permit number: AU-COM2012-171.

Mean monthly water-level data at the South Alligator River Data Warehouse (12°39´42´´S, 132°30´26´´E) from 1979 to 2011 was obtained from the Department of Land Resource Management Water Data Portal < http://www.lrm.nt.gov.au/water/water-data-portal>. These data were used to broadly characterize water-level variation at the study area.

### Fish and primary resources sampling

We sampled 13 water bodies, including waterholes and floodplains, but none of those waterbodies were visited during both dry and wet seasons because of lack of water or access. Nevertheless, most waterbodies sampled during the dry season were represented by their surrounding floodplains during the wet season (Fig 1). We concentrated sampling during the late-dry (October-November, 2013) and late-wet seasons (April-May, 2014).

Mullet were caught mainly using gill nets. Electrofishing was used in a few waterholes during dry-season sampling. Fish were measured (Standard length, ±1 mm), weighed (±1 g) and dissected to collect samples of muscle, liver, gonads and eggs, when present. We placed all tissue samples in labeled cryogenic vials and stored them immediately on ice for SIA and in liquid nitrogen for RNA:DNA analyses. All animals were euthanized using clove oil, and all efforts were made to minimize suffering. Only 42 of the 56 individuals collected were of adult size (standard length > 27 cm), and juveniles were not used in analyses.

The use of animals in this study was approved by Griffith University’s Animal Ethics Committee in accordance with the Australian Government’s code for the care and use of animals for scientific purposes. Permit Number: ENV/08/11/AEC "NABH-Northern Australia Biodiversity Hub".

We included data of other components of aquatic foodwebs, including biofilm, detritus and filamentous algae as possible primary resources for fish (S2 Table). These data were collected as part of a broader foodweb project between 2012 and 2014. The samples of these primary sources were collected in ten of the same sites where mullet were captured, and samples from 11 additional sites were also included in analyses (Fig 1). Specific methods used to collect end-member organisms are summarized in [49].

### RNA:DNA laboratory processing

On arrival at the laboratory, samples were removed from liquid nitrogen and kept frozen at -80°C for a maximum of 15 days. We randomly took sub-samples weighing between 0.001 and 0.3g for analyses. We added 200μl of 0.5% Sarcosil-TE buffer (0.5% sarcosyl; 10mM Tris-HCl, pH 7.5; 1mMEDTA) and two sterile beads (3mm) to the samples in order to induce cell lysis by high-frequency oscillation. RNA and DNA content were quantified using a QubitTM flourometer and fluorescent dyes and standards from Qubit^®^ dsRNA—DNA BR Assay Kits. RNA and DNA content were expressed as μg/mL. RNA:DNA represents the ratio of these concentrations.

### SIA Laboratory processing

In the laboratory, all samples were kept frozen at -20°C for 2–4 weeks. We then dried the samples in an oven at 60°C for at least 24 h before grinding and homogenizing them with a mortar and pestle. Samples of 0.6–1.0 mg were used in the analyses.

Samples were combusted in a EuroEA 3000 (EuroVector, Italy) or Europa GSL (Sercon Ltd, Crewe, UK) elemental analyzer and the resulting N_2_ and CO_2_ gas were chromatographically separated and fed into an IsoPrime (Micromass,UK) or Hydra 20–22 (Sercon Ltd, Crewe UK) isotope-ratio mass spectrometer. This measures the ratio of heavy and light isotopes in a sample and compares them to a standard. Elemental ratios (C/N) are expressed in %C and %N by mass and isotope ratios (*δ*) as parts per mil (‰), defined as *δ*(‰) = (R_sample_/R_standard_—1)*1000, where R_sample_ and R_standard_ are the isotope ratios of the sample and standard, respectively. Isotopic standards used were referenced to PeeDee Belemnite (PDB) for carbon, and atmospheric air for nitrogen [32]. Secondary standards of Ammonium Sulfate and Sucrose were used in each run. Acetanilide was used to cross reference elemental compositions of secondary standards.

### Data analysis

We used exploratory bi-plots and regressions to examine relationships between length and body mass, and used regression residuals as an index of body condition. Because high C/N ratios in animal tissues are indicative of high lipid content [50–52], we used C/N as a secondary indicator of condition. Also, because high lipid levels can cause isotopic fractionation when C/N is higher than 4.0 [50], we performed chemical lipid extractions on a subset of 29 samples (10 muscle, 9 gonads and 10 liver) using a chloroform:methanol solution following the protocol from Bligh and Dyer [53]. After re-analyzing these samples for stable isotopes, we compared the resulting *δ*^13^C values with those mathematically lipid corrected using C/N and the most common equations in the literature [51, 52, 54–57]. We used the slopes and fit (r^2^) of the relationships to choose the most appropriate equation to correct the remainder of the samples. The equation from [51] yielded results that best matched values from our extracted samples, so we mathematically corrected samples with C/N ratios ≥4.0 using that equation (S1 Fig).

We explored the relationships between reproductive and somatic tissues in terms of RNA:DNA ratios and carbon and nitrogen stable isotopes using simple and multiple linear regressions. Specifically, we tested whether *δ*^13^C and *δ*^15^N of reproductive tissues (gonads) were predicted by somatic tissues (muscle and liver) and also whether long-term (muscle) is predicted by short-term turnover tissue (liver).

To test for differences between dry and wet seasons on carbon and nitrogen SI of mullet tissues (muscle, liver and gonads) and primary resources (detritus, filamentous algae and biofilm), we used one-way Analyses of Variance (ANOVA).

We used a two-way ANOVA to compare RNA:DNA means using season and tissue type as factors. We used Analyses of Covariance (ANCOVA) to test if gonadal RNA:DNA ratios are related to gonadal *δ*^15^N and season.

We also included the site of capture nested within seasons as factor to control for confounding effects of different locations being sampled. R software [58] was used for all statistical analyses and graphics.

## Results

We found no evidence suggesting that the site of capture influenced any of the patterns explained regarding body condition, RNA:DNA ratios and *δ*^15^N of different tissues (F<2.4; p>0.067 in all cases). However, we found a significant influence of the site where mullet were caught on *δ*^13^C of sampled tissues (F = 2.8; p = 0.022), suggesting site-specific *δ*^13^C signatures being incorporated on mullet tissues. During the wet season, most individuals had immature or early developing gonads. During the dry season, most had early developing gonads, but four had ripe eggs. There was a significant positive correlation between standard length and body mass (log(y) = -5.13 + 3.18*log(x); r^2^ = 0.69; p<0.001). During the wet season, most individuals were heavier for a given length, and conversely during the dry season they were lighter than expected for their length (S2 Fig). We used the residuals from the length—body mass regression as an index of condition.

**Fig 2:**
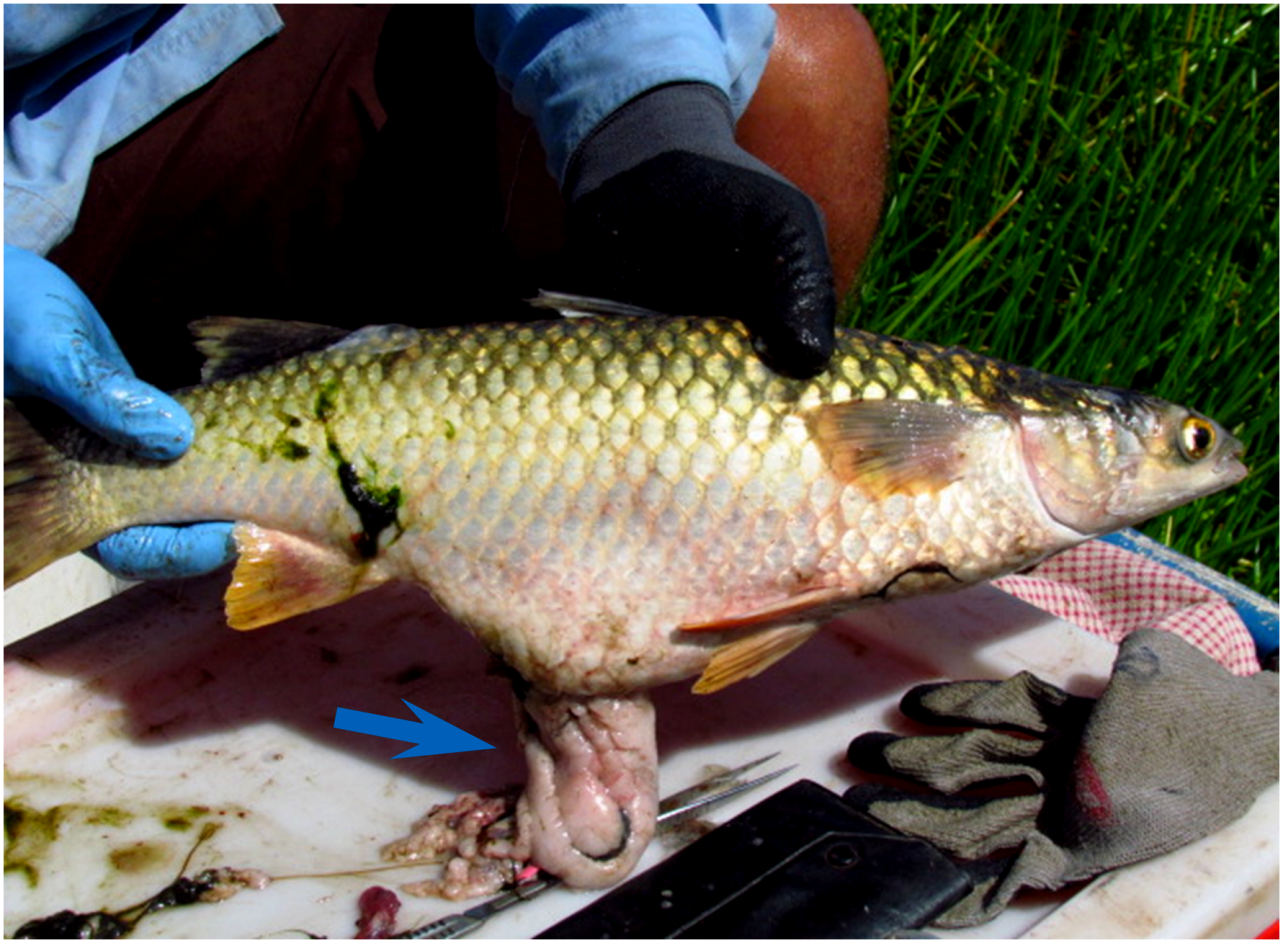
Adult individual of *Liza alata*. During the wet season, individuals of this species possess large mesenteric fat bodies (blue arrow) representing up to one third of the body volume.

Significant differences in body condition were found between seasons (t-test; t = -8.91, df = 33, p< 0.001), and all individuals had better body condition during the wet season. Although not quantified, we observed the presence of large mesenteric fat bodies comprising around one third of body volume in individuals during the wet season (Fig 2).

**Fig 3:**
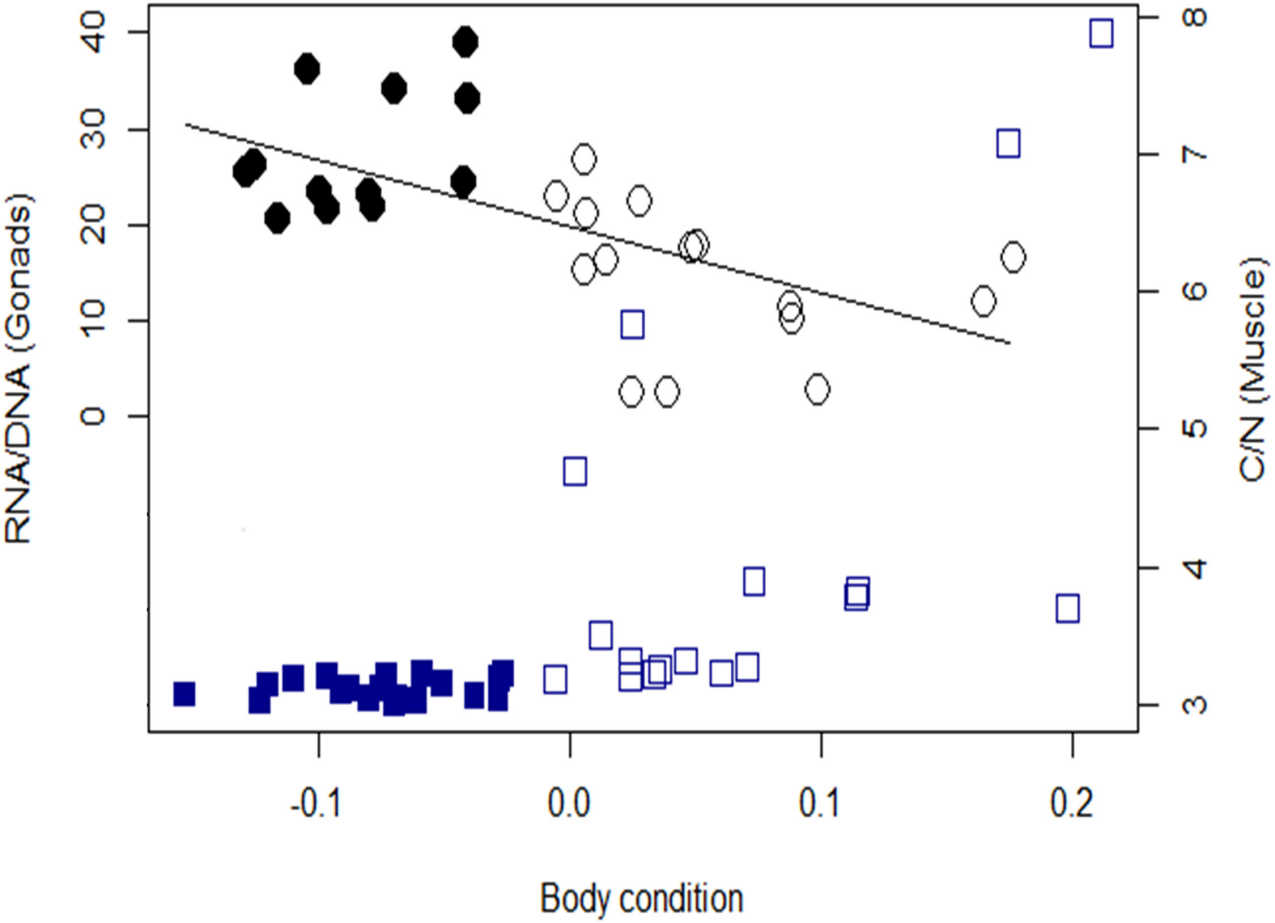
Relationships between body condition, reproductive investment and lipid content in somatic tissues. Regression residuals of standard length and body mass were used as surrogates of body condition (X axis). The Y axis represents RNA:DNA ratios in gonads, a proxy of reproductive investment (black circles). The Z axis represents C/N ratios, a proxy of lipid content in muscle tissue (blue squares). Individuals from dry and wet seasons are represented by solid and open symbols, respectively.

There was a strong positive, though non-linear, correlation between C/N ratios of muscle tissue and the regression residuals, suggesting that the two indicators were in good agreement, and C/N ratios were also higher during the wet season (t = -2.86, df = 16.1, p = 0.011) (Fig 3).

When data from wet and dry seasons were combined, body condition (BC) predicted RNA:DNA (R/D) in gonads (R/D = 21.02–63.5 BC, F = 15.8, r^2^ = 0.36, p<0.001). R/D was negatively related to BC, suggesting a trade-off between reproduction and somatic investment throughout the year. However, this relationship was not evident when analyzing each season separately (Dry season: r^2^ = 0.11, p = 0.159; Wet season: r^2^ = 0.04, p = 0.222) (Fig 3).

**Fig 4:**
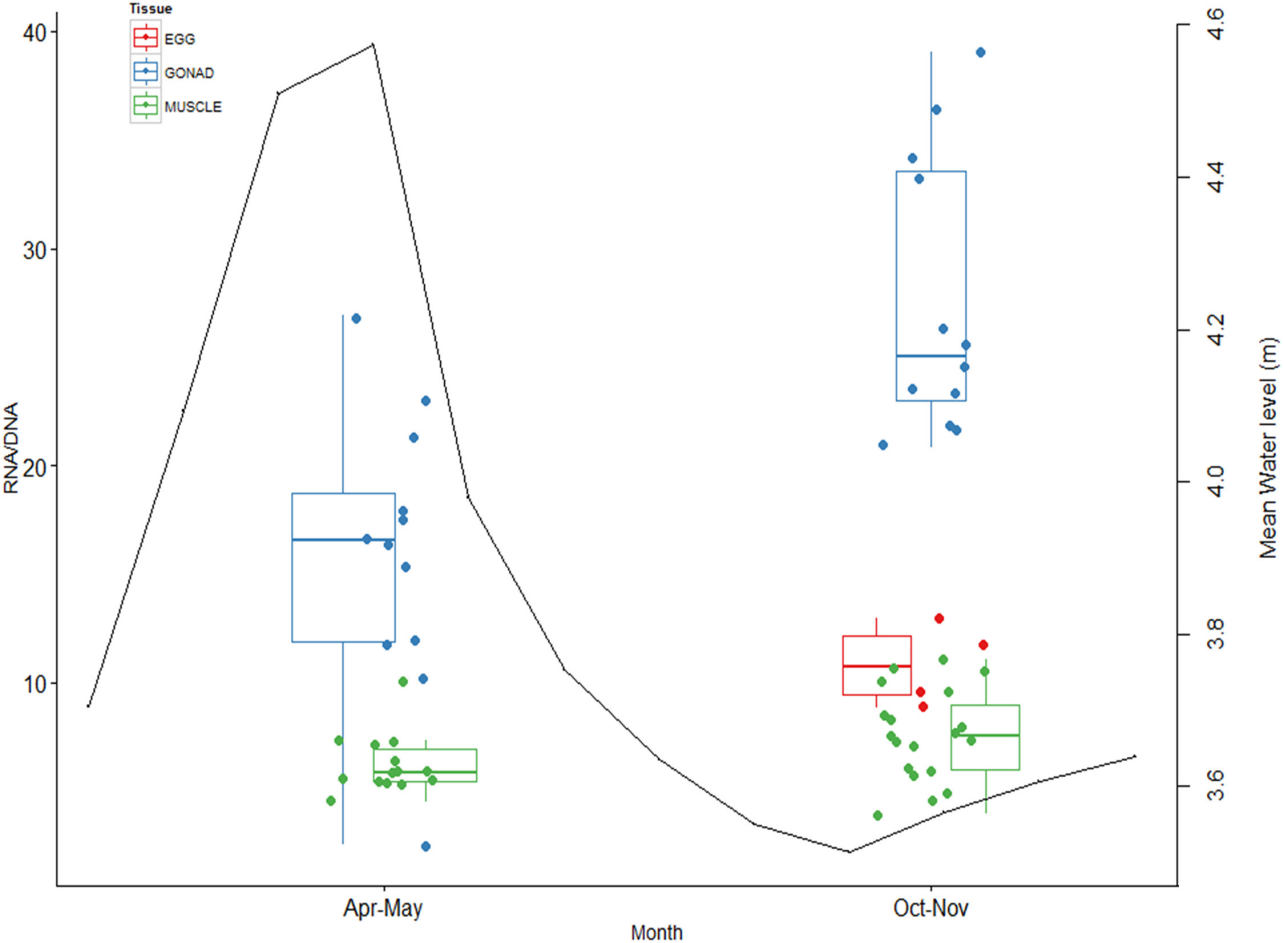
Somatic and reproductive growth. Growth of tissues is indicated by RNA:DNA ratios in relation to the flooding cycle (mean monthly water level data at the South Alligator River Data Warehouse, black line). Developed eggs, gonads and muscle tissues are represented by red, blue and green dots, respectively.

A two-way ANOVA on RNA:DNA values showed a significant interaction between season and tissue type (F = 18.93, p<0.001), suggesting that despite the lower body condition and lipid levels, most growth occurred in gonads during the dry season, when water level was at its lowest (Fig 4 and Table 1).

**Table 1:** RNA:DNA values of *Liza alata* tissues.

	Dry		Wet	
Tissue	Mean (n)	± SD	Mean (n)	± SD
EGGS	10.83 (4)	1.92	-	-
GONAD	27.54 (12)	6.39	14.00 (12)	7.83
MUSCLE	7.61 (19)	2.11	6.22 (14)	1.27

Means and standard deviations (± SD) of RNA:DNA values from different tissues during dry and wet seasons.

No significant differences in *δ*^13^C and *δ*^15^N of mullet tissues and primary resources (filamentous algae and biofilm) were found between dry and wet seasons. However, detritus *δ*^15^N was 2.3‰ more enriched during the wet season (Table 2).

**Table 2:** Stable isotopes of C and N of *L*. *alata* and its primary sources.

	*δ*^13^C (±SD)				*δ*^15^N (±SD)			
	Dry	Wet	F	p	Dry	Wet	F	p
*L*. *alata* (M)	-29.02 (±1.81)	-29.83 (±2.85)	1.08	0.304	7.11 (±0.77)	6.91 (±0.99)	0.5	0.484
*L*. *alata* (G)	-28.44 (±2.03)	-29.32 (±2.28)	1.33	0.258	5.45 (±0.77)	5.82 (±1.06)	1.24	0.274
*L*. *alata* (L)	-28.68 (±2.35)	-30.25 (±2.80)	3.46	**0.071**	6.50 (±1.04)	6.45 (±0.71)	0.03	0.854
Detritus	-31.26 (±0.66)	-30.28 (±3.37)	1.06	0.322	1.39 (±1.31)	3.71 (±2.53)	4.39	0.058*
Filam. algae	-28.47 (±5.15)	-35.69 (±1.33)	3.71	**0.072**	2.95 (±2.20)	2.4 (±2.74)	0.11	0.746
Biofilm	-27.03 (±2.97)	-26.19 (±3.46)	1.37	0.244	4.33 (±2.97)	3.71 (±2.0)	0.61	0.436

Mean and Standard deviation (±SD) values of carbon (*δ*^13^C) and nitrogen (*δ*^15^N) stable isotopes from mullet tissues (M = muscle, G = gonad, L = liver) and their main primary sources available during dry and wet seasons. F and p values correspond to results from a One-Way ANOVA testing differences on *δ*^13^C and *δ*^15^N between seasons.

Carbon stable-isotope ratios (*δ*^13^C) of somatic and reproductive tissues were highly correlated. Liver *δ*^13^C significantly predicted both gonad (*δ*^13^C_G_ = -8.55 + 0.69*δ*^13^C_L_, F = 62.9, r^2^ = 0.65, p<0.001) and muscle (*δ*^13^C_M_ = -12.08 + 0.57*δ*^13^C_L_, F = 30.1, r^2^ = 0.46, p<0.001) (S3A and S3B Fig, respectively). Gonad *δ*^13^C was also predicted by muscle *δ*^13^C (*δ*^13^C_G_ = -5.91 + 0.79*δ*^13^C_M_, F = 49.1, r^2^ = 0.59, p<0.001) (S3C Fig). Standardized regression coefficients from a multiple linear regression model (R^2^ = 0.72, p<0.001) indicated that the variation in *δ*^13^C from gonads was equally predicted by the variation in liver (b_standardized_ = 0.43, p<0.001) and that in muscle (b_standardized_ = 0.43, p = 0.003), but season had no significant effect (b_standardized_ = -0.19, p = 0.64).

**Fig 5:**
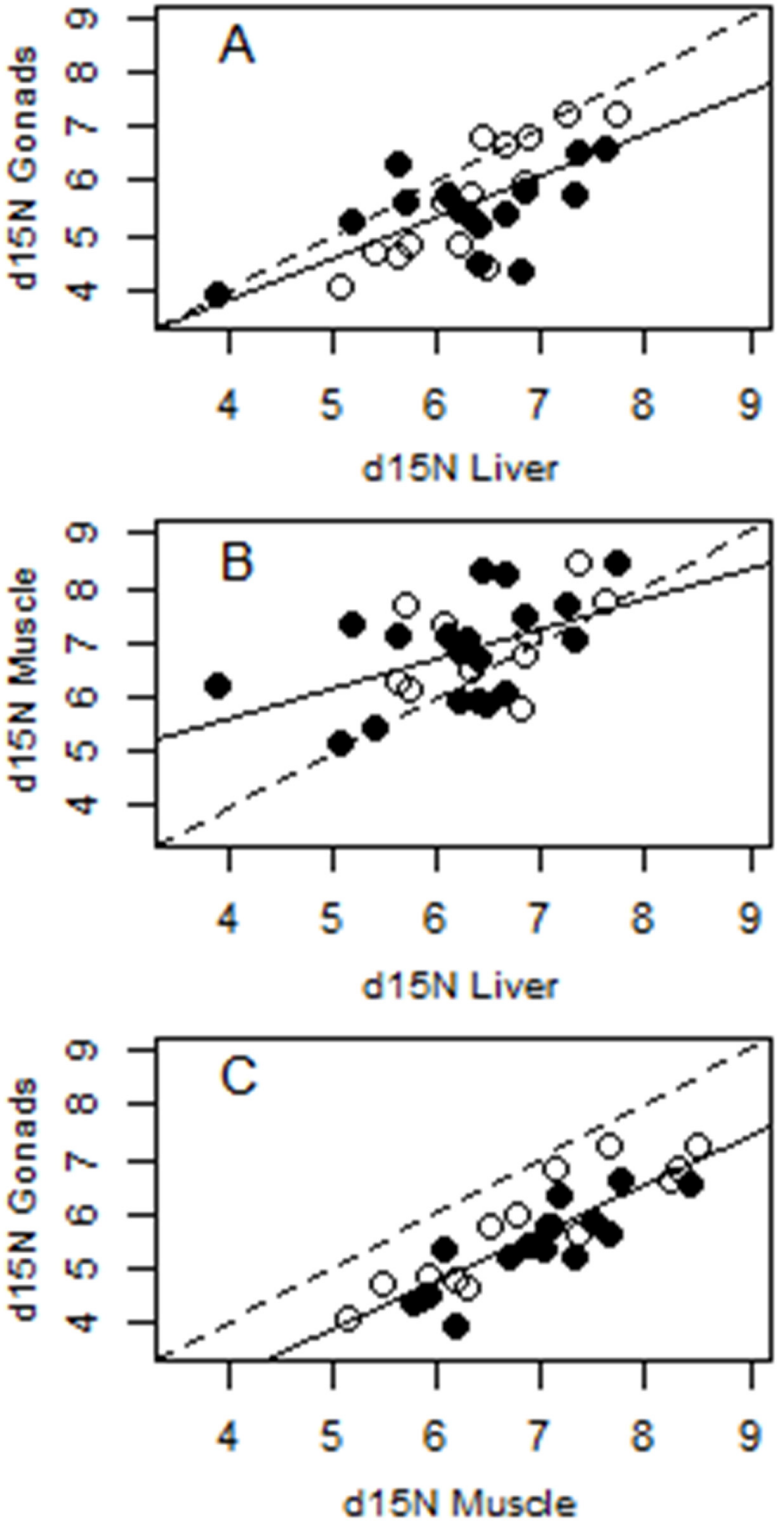
Relationships between *δ*^15^N values in somatic and reproductive tissues of *Liza alata*. The dashed line represents the 1:1 expected relationship. The solid line represents the least-squares linear regressions of: A) liver vs. gonads, B) liver vs. muscle and, C) muscle vs. gonads. Individuals from dry and wet seasons are represented by solid and open symbols, respectively.

Nitrogen stable isotopes from liver (*δ*^15^N_L_) had a relatively weak relationship with gonads (*δ*^15^N_G_ = 0.78 + 0.76 *δ*^15^N_L_, F = 21.01, r^2^ = 0.43, p<0.001) and muscle (*δ*^15^N_M_ = 3.4 + 0.55 *δ*^15^N_L_, F = 9.08, r^2^ = 0.22, p = 0.005) (Fig 5A and 5B, respectively), but the relationship between muscle and gonad was stronger (*δ*^15^N_G_ = -0.55 + 0.89 *δ*^15^N_M_, F = 80.43, r^2^ = 0.74, p<0.001). Gonads were consistently lower in *δ*^15^N values relative to muscle, which caused the relationship to fall below the 1:1 line (Fig 5C). Standardized regression coefficients from a multiple regression model (*δ*^15^N_G_ = -3.16 + 8.24_*δ*_^15^_NM_+ 3.62_*δ*_^15^_NL_ + 3.08_Season_, R^2^ = 0.85, p<0.001) indicated that variation in *δ*^15^N in gonads was better predicted by *δ*^15^N in muscle (b_standardized_ = 0.73, p<0.001) than that in liver (b_standardized_ = 0.32, p = 0.001) or by season (b_standardized_ = 0.4, p = 0.004).

Analysis of covariance indicated that RNA:DNA ratios were significantly related to *δ*^15^N in gonads (p = 0.04), and season (p = 0.02), but there was no interaction between season and *δ*^15^N (p = 0.09).

## Discussion

Using a novel combination of chemical indicators, we found evidence of temporal uncoupling between resource availability and allocation of energy to reproduction by *Liza alata* in the Alligator Rivers region. Fish were in better condition in the wet season when food availability peaks, yet were actively synthesizing gonads in the dry season. A trade-off between reproductive and somatic investment was evident and carbon and nitrogen stable isotopes suggested that the long-term diet was mainly contributing to reproductive growth.

We used the residuals from the length-mass relationship of mullet as an index of body condition. A strong correlation between a second indicator of condition, C/N ratios of muscle tissue, and the regression residuals of length and body mass suggests that residuals can appropriately be used as an index of body condition. The relationship between C/N and the regression residuals is likely nonlinear because, in addition to storing fat in muscle, this species also stores fat in specialized mesenteric fat bodies. Therefore, although they may be confounded by reproductive tissue or stomach fullness, indices based on length-weight relationships appear more sensitive than muscle C/N ratios for assessing fish condition.

Although mullet in this study showed evidence of gonadal growth during both dry and wet seasons, RNA:DNA ratios imply that higher growth occurred during the dry season. This was unexpected because the dry season is the period of the year when water level is at its lowest, as is primary productivity and food availability in the Alligator Rivers region [7]. Given the high prevalence of mesenteric fat bodies and the higher lipid content of muscle (higher C/N) in fish captured during the wet season, this suggests that mullet store most of their energy for reproductive and somatic growth as fat when resources are more abundant and they can attain a favorable body condition. The fat is then re-mobilized to the gonads during the dry season, reducing condition but increasing gonad mass in preparation for spawning in the early-wet season.

Although RNA:DNA values of muscle tissue were slightly higher during the dry season, this tissue grew significantly less relative to gonads during both seasons. Slow growth rates, a common characteristic of related species within the Mugilidae [59], might be causing this pattern. Some Mugilids attain about 75% of their maximum size in their first 3–4 years of life, with greatest mean annual growth increments during the first year and decreasing markedly after age 3–5 [60]. All mature mullet analyzed in this study had standard lengths between 27 and 42 cm. It is plausible that these individuals were growing slowly and with limited food consumption rates outside of the wet season.

Furthermore, low values of RNA:DNA found in developed eggs might be the result of increasing volumes of lipid granules during pre-ovulatory stages, typical of other Mugilidae species [61]. Throughout oogenesis, early oocytes are rich in protein and RNA within the yolk nucleus. Subsequently, with the approach of the breeding season, during the vitellogenic period, lipid droplets accumulate in the cytoplasm [61]; this might explain such low values of RNA:DNA in developed eggs relative to gonads.

Life-history theory states that organisms have a limited resource budget and thus, allocation to reproduction arises as a trade-off against somatic growth or survival. The "Principle of Allocation" [62] predicts negative correlations between reproduction and somatic growth [9–14]. Somatic investment is usually used as a surrogate of body condition (e.g. fat content or body mass per length) [14]. The strong negative correlation between body condition and RNA:DNA in mullet gonads in this study provides evidence of a trade-off between reproduction and somatic investment. Although expected, this trade-off has rarely been measured in natural conditions, mainly because individual variation in resource acquisition exceeds that of resource allocation [15]. Estimates of reproductive investment in most studies under natural conditions rely on values such as total clutch mass, the number of young in a clutch and frequency of clutches (see [14]). However, there are difficulties associated with quantifying fecundity or reproductive effort of fish based on counting eggs [8] because there could be confounding effects, such as downregulation of secondary oocytes by atresia which may reduce the final number of eggs ovulated [63–66]. Since RNA:DNA ratios are molecular measures of instantaneous growth of specific tissues, such as gonads, they are likely to be useful estimates of reproductive investment. This can overcome the difficulties of estimating reproductive effort, especially in capital breeding fishes.

Because capital breeders [67–69] use stocks of energy in their body to sustain reproduction, a positive correlation between reproductive investment and pre-breeding body stores is expected [14]. This is true in the case of mullet in this study. Low values of gonadal RNA:DNA during the period when body condition is at its highest suggest that the wet season is the time of the year when mullet store most energy. On the other hand, elevated values of gonadal RNA:DNA ratios during the dry season suggest that these energy stocks are allocated to reproduction months after they were acquired. In combination, these lines of evidence suggest that *L*. *alata* is a capital breeding fish which shows a temporal uncoupling of resource ingestion, energy storage and allocation to reproduction.

Further evidence for capital breeding is provided through carbon and nitrogen stable isotope analyses. Isotopic equilibrium depends on turnover rates of tissues, and recent dietary sources are more rapidly reflected in fast-turnover tissues such as liver [37–40]. Diamond mullet in this study showed strong correlations of *δ*^13^C values in reproductive (gonads) and somatic tissues (liver and muscle), suggesting that both short- and long-term diet could be contributing to gonadal growth. If gonads were being synthesized using only energy stored months before when mullet had access to resources from floodplains, then we would expect *δ*^13^C values of gonads to be poorly correlated with those of liver and more correlated with tissues showing slower turnover rates, such as muscle. However, we also found a significant positive correlation between *δ*^13^C values for liver and muscle tissues. This strong correlation may be due to overall isotopic similarity between wet and dry season habitat resources. Although filamentous algae showed a slight increase in *δ*^13^C values which might explain the slight increase in liver *δ*^13^C values during the wet season, we found no significant differences between dry and wet season *δ*^13^C values of other potential mullet resources, such as detritus and biofilm.

Results of *δ*^15^N analyses were more consistent with expectations for a capital breeder. Although gonad *δ*^15^N values showed significant correlations with liver values, a stronger correlation was found with muscle tissue, as revealed by a significantly greater slope and r^2^. Higher *δ*^15^N in muscle tissue that turns over more slowly than in fast-turnover liver tissue might be a reflection of the significantly higher *δ*^15^N found in detritus in the wet season. Detritus is an important resource for this species and during the wet season, mullet forage in more productive floodplains than during the dry season. It has been observed that high denitrification processes in wetland areas cause primary consumers to have higher *δ*^15^N isotopic values than in areas with less wetland coverage [70].

The low correlations between *δ*^15^N of muscle and gonads (likely produced during wet season) with liver (produced in less productive areas during the dry season) suggests that N-bearing proteins in gonads may be more readily mobilized from protein obtained during the wet season than sourced from a maintenance diet during gonadal formation in the dry season. Although there was a strong correlation between gonads and muscle tissue, gonads showed consistent depletions in ^15^N relative to muscle. Furthermore, we found that instantaneous reproductive growth (gonadal RNA:DNA) was negatively correlated with *δ*^15^N values during the dry season. Both of these patterns are consistent with results obtained by [71] for humans, who demonstrated that *δ*^15^N values of hair can become low due to anabolic processes, such as those occurring during gestation. The authors hypothesized that higher retention of urea might be helping to re-incorporate N to the metabolic pool for protein synthesis. However, fish excrete N in the form of ammonia which is highly toxic, which makes it unlikely that mullet would be retaining and recycling excretory N. In general, N cycling is complex and can lead to enrichment or depletion depending on the tissue and physiological state of the organism (see review by [72]), and the amino acid profile of different tissues can also affect *δ*^15^N because essential amino acids such as phenylalanine exhibit no fractionation relative to the diet whereas others such as glutamic acid exhibit strong fractionation [73]. Detailed physiological explanation for ^15^N depletion in mullet gonads is beyond the scope of this study, but a subject worthy of future investigation. To our knowledge, this is the first record of ^15^N depletion in fish tissues related to energy allocation to reproduction.

Although the sites where mullet were captured during the wet season were not exactly the same as those during the dry season because of lack of access or water availability, respectively, it is important to stress that the patterns found relating somatic and reproductive growth and *δ*^15^N of mullet tissues were not affected by these differences. On the other hand, *δ*^13^C values of mullet tissues were significantly influenced by the site of capture, this suggests that site-specific *δ*^13^C signatures are being incorporated on mullet tissues. This is an expected result given that *δ*^13^C values of primary aquatic producers depend mainly on CO_2_ difusion rates and local isotopic composition of the dissolved inorganic carbon pool (DIC) [74].

The temporal and spatial uncoupling between energy acquisition and allocation to reproduction of this common fish has important implications for the preservation of the natural hydrological regimes of floodplain areas. Diamond mullet typically inhabit waterholes during the dry season and move into floodplains during the wet season where they may obtain most of their energy. Subsequently, as flood waters recede, some individuals migrate back to waterholes and others eventually migrate to saltwater to spawn [23]. Despite the low primary productivity found in remnant waterholes during the dry season [7], findings from this study emphasize the importance of these habitats as zones where reproductive allocation takes place. More importantly, wet season habitats such as floodplains are critical in providing most energy for growth and reproduction. Therefore, the maintenance of natural hydrological regimes would enhance the capacity for fishes of this region to maintain viable populations.

## Supporting Information

S1 Fig Relationships between lipid-extracted and mathematically-corrected *δ*^13^C data using published lipid-correction equations. The X axis represents mathematically-corrected (*δ*^13^C´) minus uncorrected (*δ*^13^C) values. The Y axis represents chemically-extracted (*δ*^13^C_ext_) minus uncorrected (*δ*^13^C) values. Gonad, liver and muscle tissues are represented by black dots, triangles and squares, respectively. The dashed line represents the expected 1:1 relationship. The solid line represents a least squares regression of *δ*^13^C_ext_—*δ*^13^C on *δ*^13^C´- *δ*^13^C. The correction equations used were as follows: **(A)** [47]: *δ*^13^C´ = *δ*^13^C - 2.98*log(C/N) + 3.09; **(B)** [50]: *δ*^13^C´ = *δ*^13^C +(6-(22.2/C/N)); **(C)** [51]: *δ*^13^C´ = (*δ*^13^C*C/N + 7.08*(C/N-3.7)) / C/N; **(D)** [52]: *δ*^13^C´ = *δ*^13^C + (0.322* C/N) - 1.175; **(E)** [48]: *δ*^13^C´ = *δ*^13^C - 3.32 + (0.99* C/N); **(F)** [53]: *δ*^13^C´ = *δ*^13^C + (6.3* ((C/N—4.2) / C/N)).
(TIF)

S2 Fig Linear regression between log standard length and log body mass of *Liza alata*. Individuals captured during dry and wet seasons are represented by solid and open symbols, respectively.
(TIF)

S3 Fig Relationships between *δ*^13^C values in somatic and reproductive tissues of *Liza alata*. The dashed line represents the 1:1 expected relationship. The solid line represents the least-squares linear regressions of: A) liver vs. gonads, B) liver vs. muscle and, C) muscle vs gonads. Individuals from dry and wet seasons are represented by solid and open symbols, respectively.
(TIF)

S1 Table Data of underlying findings. Raw data of the individuals of *L*. *alata* from which we draw the conclusions presented in the manuscript. Abreviations of attributes are as follows: sl_mm = standard length in milimeters, mass_g = mass in grams, %c = percentage of carbon, %n = percentage of nitrogen, le = lipid-extracted samples, d13c = *δ*^13^C, d15n = *δ*^15^N, r_d = RNA:DNA ratio, M = muscle, L = liver, G = gonad, EGG = eggs. Sex: F = female, M = male, U = unknown.
(XLS)

S2 Table Primary sources data of underlying findings. Raw data of the primary sources (biofilm, detritus and filamentous algae) from which we draw the conclusions presented in the manuscript. Abreviations of attributes are as follows: d13c = *δ*^13^C, d15n = *δ*^15^N, %c = percentage of carbon, %n = percentage of nitrogen.
(XLS)
