# Supplementary figures and images for: Temporal Uncoupling between Energy Acquisition and Allocation to Reproduction in a Herbivorous-Detritivorous Fish
Source: PLoS One. 2016 Mar 3;11(3):e0150082. doi: 10.1371/journal.pone.0150082 (PMC4777362; doi:10.1371/journal.pone.0150082)

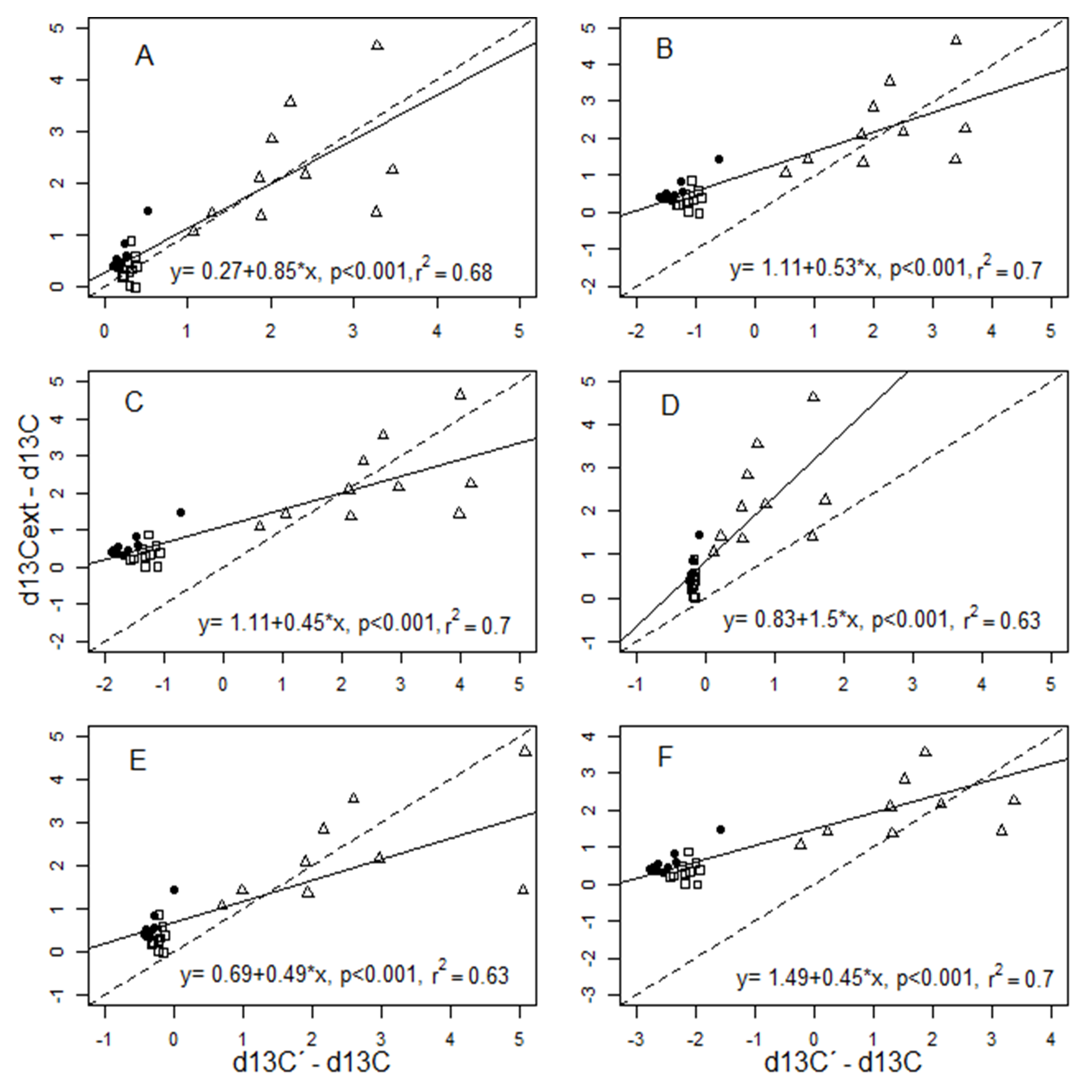

Supplement: S1 Fig — The X axis represents mathematically-corrected (δ13C´) minus uncorrected (δ13C) values. The Y axis represents chemically-extracted (δ13Cext) minus uncorrected (δ13C) values. Gonad, liver and muscle tissues are represented by black dots, triangles and squares, respectively. The dashed line represents the expected 1:1 relationship. The solid line represents a least squares regression of δ13Cext—δ13C on δ13C´- δ13C. The correction equations used were as follows: (A) [47]: δ13C´ = δ13C - 2.98*log(C/N) + 3.09; (B) [50]: δ13C´ = δ13C +(6-(22.2/C/N)); (C) [51]: δ13C´ = (δ13C*C/N + 7.08*(C/N-3.7)) / C/N; (D) [52]: δ13C´ = δ13C + (0.322* C/N) - 1.175; (E) [48]: δ13C´ = δ13C - 3.32 + (0.99* C/N); (F) [53]: δ13C´ = δ13C + (6.3* ((C/N—4.2) / C/N)). (TIF) [file pone.0150082.s001.tif]

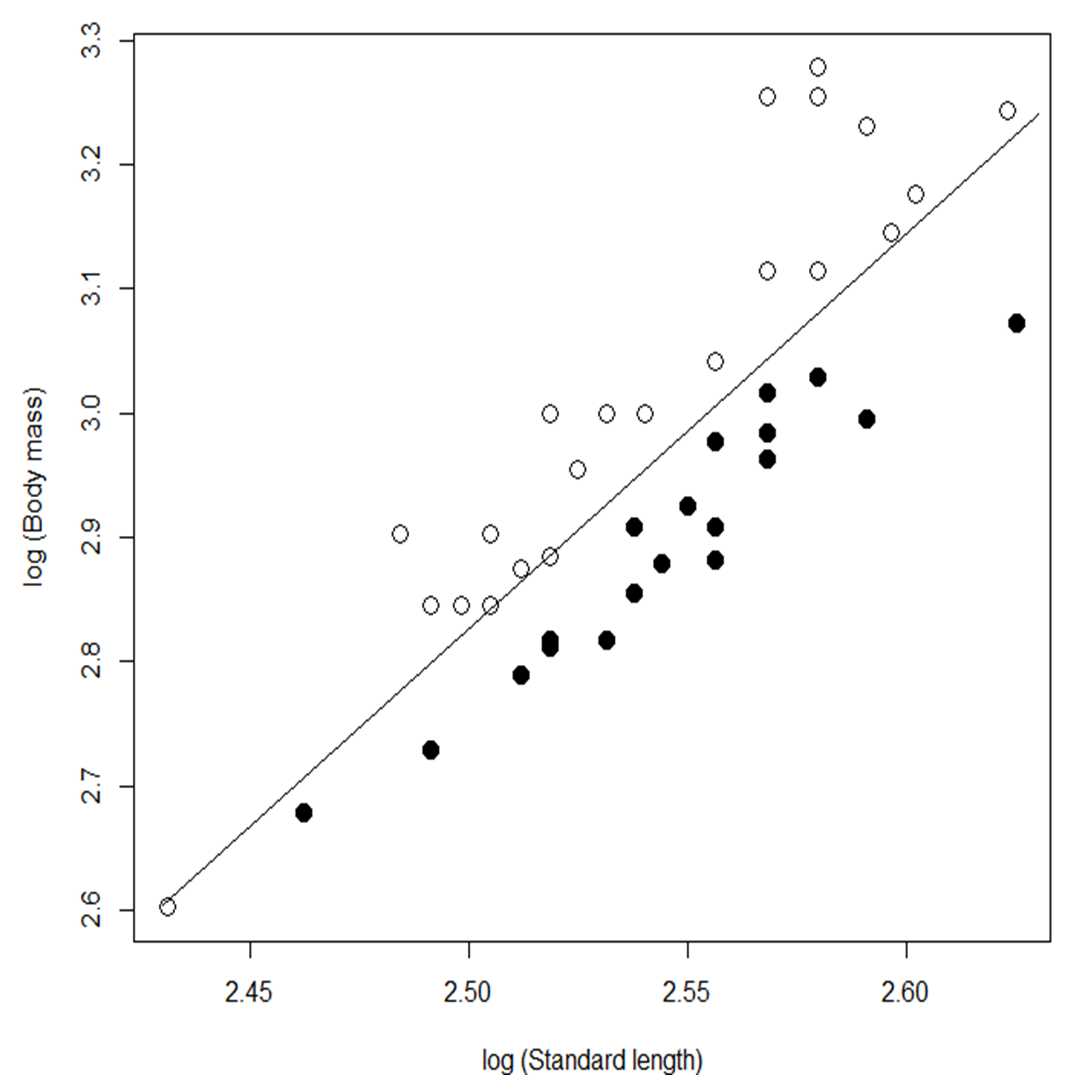

Supplement: S2 Fig — Individuals captured during dry and wet seasons are represented by solid and open symbols, respectively. (TIF) [file pone.0150082.s002.tif]

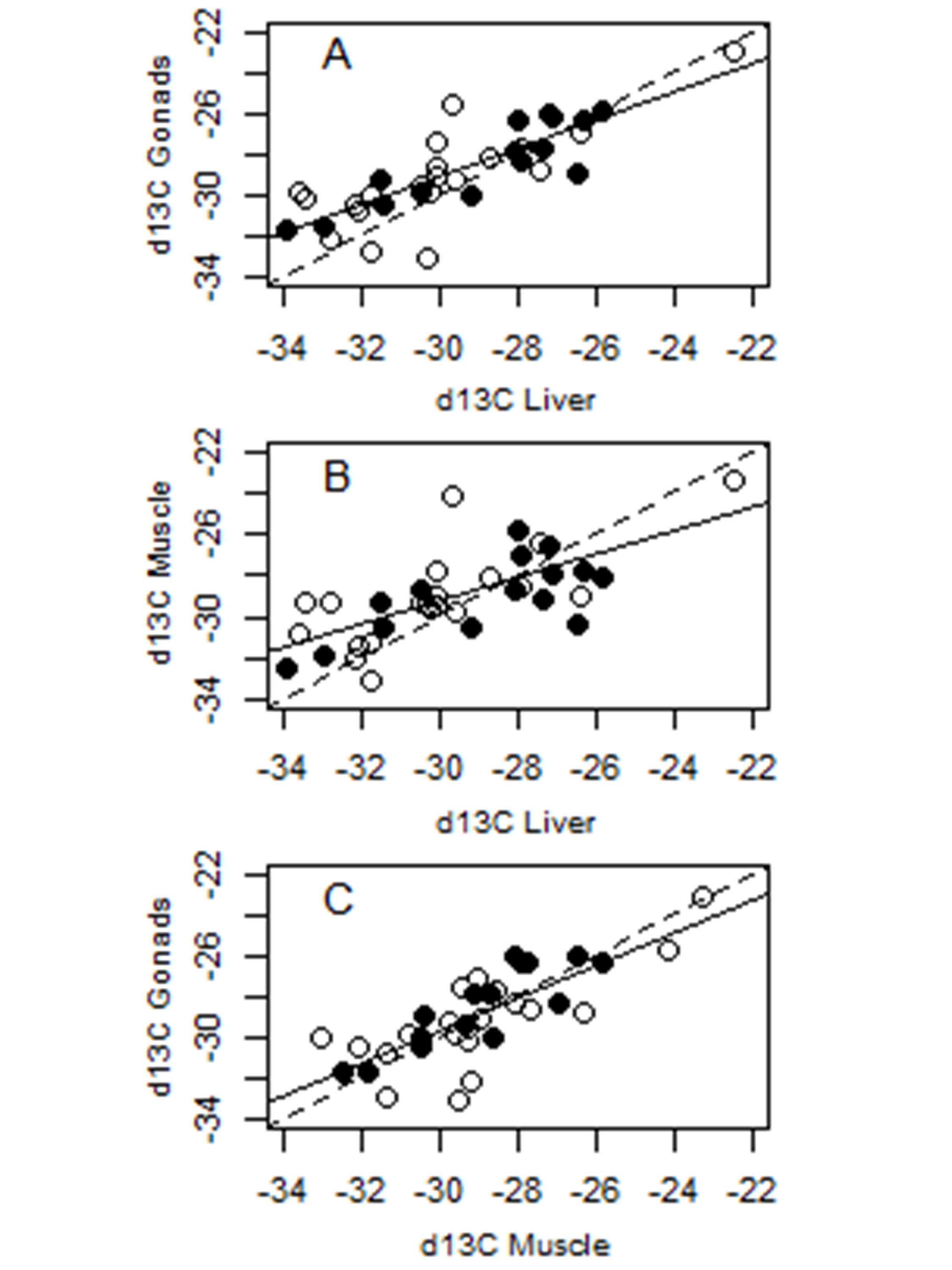

Supplement: S3 Fig — The dashed line represents the 1:1 expected relationship. The solid line represents the least-squares linear regressions of: A) liver vs. gonads, B) liver vs. muscle and, C) muscle vs gonads. Individuals from dry and wet seasons are represented by solid and open symbols, respectively. (TIF) [file pone.0150082.s003.tif]
